# Experiences of researchers with disabilities at academic institutions in the United States

**DOI:** 10.1371/journal.pone.0299612

**Published:** 2024-08-15

**Authors:** Franz Castro, Caroline Cerilli, Luanjiao Hu, Lisa I. Iezzoni, Varshini Varadaraj, Bonnielin K. Swenor

**Affiliations:** 1 Johns Hopkins Disability Health Research Center, Johns Hopkins University, Baltimore, Maryland, United States of America; 2 Lurie Institute for Disability Policy, Brandeis University, Waltham, Massachusetts, United States of America; 3 Harvard Medical School, Boston, Massachusetts, United States of America; 4 Health Policy Research Center, Mongan Institute, Massachusetts General Hospital, Boston, Massachusetts, United States of America; 5 Department of Epidemiology, Johns Hopkins Bloomberg School of Public Health, Baltimore, Maryland, United States of America; De Montfort University, UNITED KINGDOM OF GREAT BRITAIN AND NORTHERN IRELAND

## Abstract

**Introduction:**

People with disabilities are underrepresented in higher education, facing systematic obstacles such as inaccessible communication and physical environments and difficulties obtaining accommodations. This study aims to shed light on barriers to accessibility and disability inclusion in research institutions through confidential qualitative interviews with researchers with disabilities.

**Methods:**

We recruited participants via virtual flyers. Eligibility criteria included working in the United States (U.S.) as researchers that had applied for grant funding (last five years), and self-identifying as having a disability. We offered participants (total n = 35) the option of either semi-structured one-on-one live or written interviews based on their preference. Two study team members analyzed written and live responses using thematic analysis to identify themes.

**Results:**

Themes included identity/visibility, career trajectories, accessibility, accommodations, bias, representation, and inclusion. Some participants reported not disclosing their disabilities at work or during hiring processes due to fear of negative perceptions from peers or potential employers. Experiences around stigma and bias were noted both in professional relationships and when interacting with disability service offices, underscoring difficulties and delays in processes to secure accommodations. Respondents highlighted the lack of disability inclusion and low representation of people with disabilities in academia and elevated the importance of self-advocacy and of role models and mentors in shaping career pathways for future researchers with disabilities.

**Conclusion:**

Researchers with disabilities encounter systematic barriers at academic institutions, and lack of acknowledgement and research on these experiences has held back institutional and policy changes. To reduce disparities for researchers with disabilities, academic leadership must allocate resources to address ableism, create more inclusive environments, and raise standards beyond compliance with the Americans with Disabilities Act.

## Introduction

Although 21% of undergraduate students have a disability, representation of people with disabilities in higher education decreases as they progress throughout the educational pathway [1]. Consequently, people with disabilities comprise 14.7% of college graduates and 9.1% of doctorate recipients [2, 3]. People with disabilities face systematic barriers in higher education, such as inaccessible communication and physical environments [4] and difficulties obtaining accommodations [5, 6], leading to lower enrollment and graduation rates [7] and underrepresentation in the academic workforce [2]. These obstacles persist despite the Rehabilitation Act of 1973, which prohibits all programs receiving federal funding from discriminating or excluding individuals on the basis of disability [8], as well as the Americans with Disabilities Act of 1990 (ADA), which requires higher education to be made accessible to people with disabilities [9].

Brown and Leigh argue that “*ableism in academia is endemic*” [10], as the academic environment is often grounded on assumptions that cater to nondisabled people. Ableism affects disabled researchers throughout their careers, including in educational, hiring, funding, and promotion phases. Many studies have shown how institutional policies reduce access to educational opportunities in science for young adults with disabilities [6], and how the pervasiveness of these barriers throughout educational pathways negatively impacts confidence, career choices, enrollment and retention in academic programs [11, 12]. These issues persist when transitioning into the workforce; for example, scientists with early-onset disabilities who work in academic institutions receive salaries $14,360 lower than their nondisabled peers [13]. Further, there has been a decline in National Institutes of Health (NIH) grant funding for disabled researchers, demonstrating lack of support for their work [14]. This issue has received little attention, as previous research on faculty with disabilities has been excluded from higher education publications and each study is cited on average once a year [15].

Policies to increase disability inclusion in the scientific workforce in the U.S. have attracted recent attention [16]. The Biden administration issued executive orders to improve working conditions for people with disabilities in the federal workforce and to advance equity for underserved communities such as individuals with disabilities [17, 18]. Moreover, the NIH recently recognized people with disabilities as a formal health disparities population [19], and organized a working group that developed recommendations to improve the inclusion of people with disabilities in NIH-funded research studies and its research workforce [20]. These efforts have built momentum for further policy changes that support the inclusion and belonging of disabled people in research, underscoring the importance of further understanding obstacles for disabled people in research career pathways.

While previous research highlights challenges faced by students with disabilities in higher education [12, 21], few studies have examined the experiences of researchers with disabilities in the U.S. [15]. Among this work, commonly documented issues for academic staff with disabilities are navigating disability disclosure in the workplace, obtaining disability accommodations, accessing research facilities [22], facing stigma and discrimination from colleagues, and contending with structural ableism embedded within institutional policies [23].

This study uses qualitative interviews to understand barriers to accessibility and disability inclusion among U.S. academic researchers with disabilities.

## Methods

### Study design and participants

The theoretical frameworks of disability used to inform our research were the social model of disability and the human rights model of disability. The social model accounts for how researchers with disabilities are impacted by environmental factors such as ableism and social exclusion [24]. Examining shared barriers among disabled researchers through the lens of a social model allows us to understand how disparities emerge from socially created obstacles [24]. The human rights model of disability recognizes that people with disabilities should have equal rights to people without disabilities, but this community is often deprived of their rights [25]. Through the human rights model, we look into the ways policies and procedures should change to promote equal rights for researchers with disabilities. To explore the experiences of researchers with disabilities in the U.S., we recruited participants via virtual flyers followed by an online screening survey created on Qualtrics, circulated through professional networks and social media. Recruitment materials were compatible with screen reader software and included image descriptions, high-contrast, and alternative texts. The screening survey, open from March to May 2022, assessed whether participants were eligible (inclusion criteria are described below), included a brief description of the study, collected demographic data on all respondents, and asked respondents if they preferred to respond via one-on-one live virtual interviews or written interviews. We asked participants who selected one-on-one interviews to indicate any accessibility or accommodation requests, such as Communication Access Realtime Translation (CART) captioning or American Sign Language interpretation. All eligible participants were selected and contacted by a researcher for next steps via email. All participants who responded to the researcher’s follow up email were included in the sample.

Inclusion criteria for the study were having worked in the U.S. as researchers, having applied for research grant funding in the past five years, and self-identifying as having one or multiple disabilities. Using application to grant funding as an inclusion criterion allowed to better identify researchers working in academic settings instead of those working in industry. Exclusion criteria were inability to provide consent, being unable to speak English, and not providing phone or email addresses to be further contacted by researchers.

We used a broad definition of disability, including individuals who are d/Deaf, blind or have low vision, have chronic conditions, have mental illness or psychiatric disabilities, or have disabilities related to mobility (including upper and/or lower limb mobility), learning, or cognition. Opinions vary around the use of identity-first versus person-first language when referring to the disability community [26]. However, due to the diversity of participants’ responses and life experiences, we used both modalities throughout the manuscript. Study participants included researchers working in both science, technology, engineering, and mathematics (STEM) and non-STEM fields. Methods and findings are reported consistently with the Consolidated criteria for REporting Qualitative research (Document in S1 Checklist) [27]. The Johns Hopkins Medicine Institutional Review Board approved this study (#00310675).

### Data collection

Data collection took place between April and July of 2022. The interview guide, used to lead both the semi-structured one-on-one interviews and the written response prompts, included ten open-ended questions based on a literature review and lived experiences of the study team, as well as two pilot interviews with two researchers with disabilities (S1 Table). All participants provided informed consent prior to either engaging in a one-on-one interview or completing a written interview.

Participants who preferred to respond via one-on-one interviews scheduled with the interviewing researcher (LH) over email. The semi-structured one-hour one-on-one interviews were conducted over Zoom (San Jose, CA) by LH, a study team member with a disability. LH chose to disclose that she has a disability to many of the one-on-one interview participants. At the time of data collection, LH was a postdoctoral research fellow trained in qualitative research with people with disabilities. No relationships were established prior to the interview for the purposes of this study. To maximize accessibility, access requests were addressed in advance and participants were invited to engage in the way most comfortable to them, such as with their camera on or off or responding to questions verbally or by typing in the chat. Only the interviewing researcher and the study participant were present for the interviews, except in the case of the presence of American Sign Language interpreters or CART, though these individuals only served as accessibility aids and did not provide any individual input. Only audio was recorded from these interviews, and recordings were deidentified and then transcribed by a professional service (Landmark Associates, Phoenix, AZ). No field notes were recorded at the time of the interviews.

For participants who preferred to respond via written interviews, a researcher (LH) emailed the study interview questions hosted on Qualtrics (Provo, UT). Participants were given the option to type responses or to record voice messages to respond to each question, which were transcribed by researchers. No follow up interviews were conducted with any participants and no feedback was sought from participants.

### Analysis

Two study team members (FC and CC) analyzed both the one-on-one and written interview transcripts using thematic analysis at the semantic level, following Braun and Clarke’s six recursive phases of reflexive thematic analysis [28, 29]. Study team members independently generated initial codes as they first reviewed each transcript in different documents. They met regularly to produce a shared codebook and find consensus for each transcript. Disagreements were resolved by revisiting code definitions until an agreement was reached. Study team members collaboratively organized these codes into larger themes using an iterative framework to refine them to the final set of themes.

Researchers used Microsoft Word to highlight relevant text in the transcripts and typed codes using the comments functionality. The R (v 4.3.1, 2023) package *docxtractr* (v 0.6.5, 2020) was used to extract all codes from individual transcripts into.csv files. We compared these typed codes with the original codebook and found mismatches due to typos, and utilized the R package *stringdist* (v 0.9.10, 2022) to carry out approximate string matching and retrieve the correct codes. The accuracy of the matches was assessed by one of the study team members (FC).

We relied on a reflexivity approach, and we had open conversations about how our identities and experience relate to the study. At every stage of the study, at least one study team member involved identified as a person with a disability, and all study team members understood the value of disability inclusion in research. Reflexivity was done both to enrich the quality of the study through utilizing our existing knowledge bases, as well as to push to ensure we accurately represented the voices of our participants rather than filtering them through our own experiences [30, 31]. For example, the interview guide was crafted both to gather information on topics we knew were highly relevant based on our lived experience, but also phrased neutrally so as to avoid guiding participants towards only positive or negative results.

## Results

### Study population

**Fig 1:**
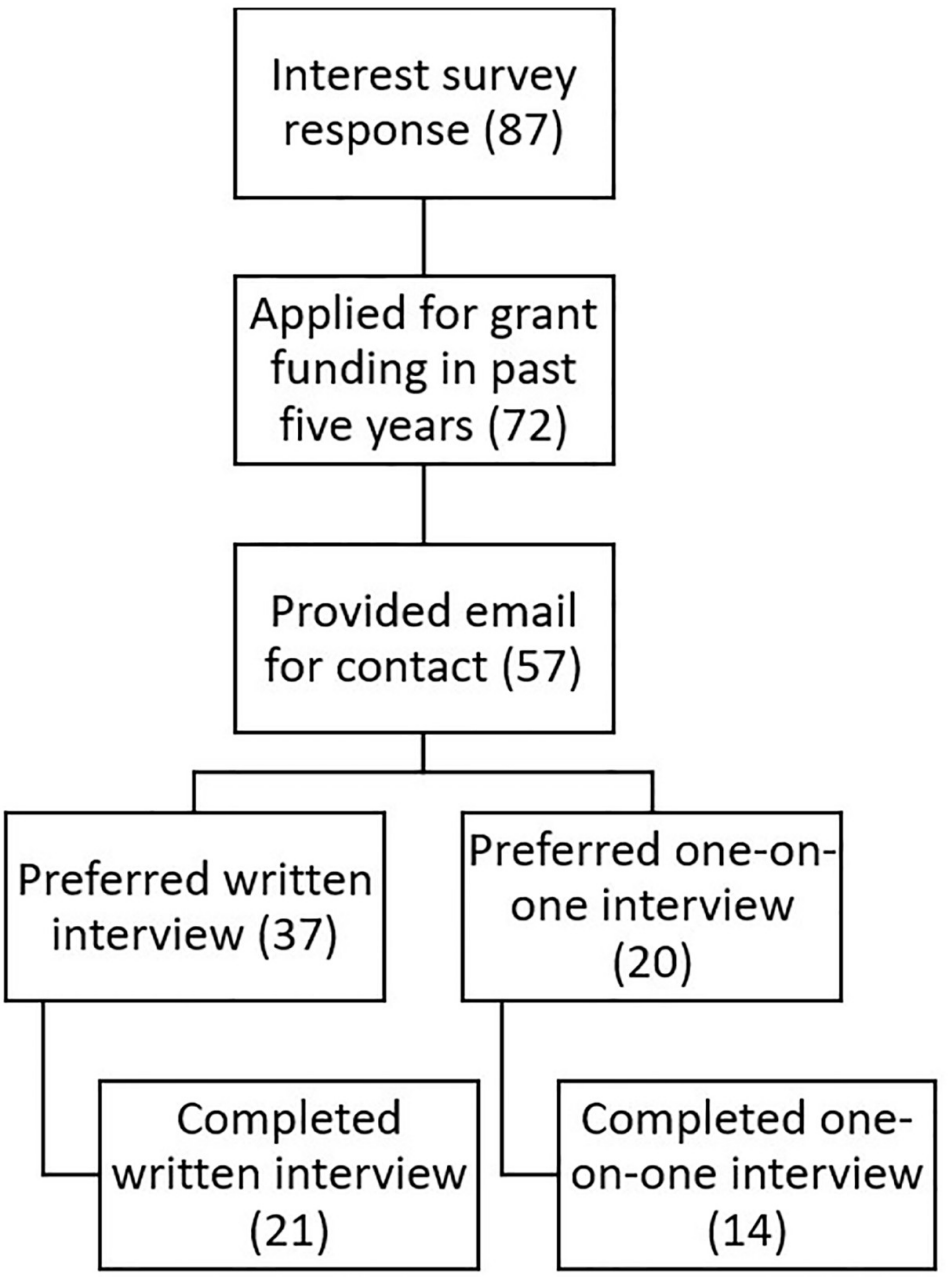
Participants’ selection and recruitment.

Eighty-seven individuals responded to the screening survey, of which 14 participated in semi-structured one-on-one interviews and 22 provided responses via written interviews, for a total of 36 responses and 35 unique participants, as one participant responded via both one-on-one interview and written interview (Fig 1). Table 1 shows respondents’ disability types; one participant is excluded from Table 1 as their response had no identifying information to link to their demographic survey response. To protect participant confidentiality, breakdown of minority reported demographic characteristics are not shared in this paper. S2 Table shows all themes and codes identified in the study and includes representative quotes for each code.

**Table 1:** Participants’ disability types.

Demographic characteristics	n (%) ^a^
Disability type	
Hearing	6 (17.1%)
Visual	5 (14.3%)
Physical or mobility	2 (5.7%)
Cognitive or intellectual	2 (5.7%)
Psychological	3 (8.6%)
Chronic or medical conditions	7 (20.0%)
Multiple / Other	10 (28.6%)

^a^ N = 35 participants.

### Identity and visibility

Nine participants reported their disabilities as apparent to others, thirteen as non-apparent, and fourteen reported that the apparentness of their disability depended on whether they were appropriately accommodated or not. Accessibility aids such as wheelchairs, hearing aids, or tinted glasses were noted as a common way that people knew their disabilities were apparent to others. Having an apparent disability was beneficial for some participants, as it quickly demonstrated to others some basic access needs, whereas other participants stated it led to further discrimination. In some cases, participants did not identify as having a disability until decades later from when they first experienced limitations. This was often related to how they felt perceived by others, how they thought their access needs and experiences compared to others, and when they received a diagnosis from a medical provider. Many people felt their disability identity was context-dependent. A participant said, “*It’s not the body that is disabling*, *it’s the intersection of a physical condition*, *deafness*, *blindness*, *whatever*, *and policies and practices that marginalize them*.” This illuminates why multiple participants were unsure whether they could identify as disabled if they had an impairment but did not experience as much stigma as they thought they needed in order to consider themselves disabled; one participant preferred instead to identify as someone with a chronic medical condition, and another reported that they felt much more comfortable identifying as disabled once they had an apparent disability. Others considered themselves disabled only when they noticed symptoms interfered with daily functioning. One individual said that after they began to identify as a person with a disability they looked back and realized that they have long made self-accommodations. Further, four participants discussed identifying as deaf or Deaf (meaning they were both deaf and involved in the Deaf community) significantly before identifying as disabled, if they did at all, as “*a large part of the Deaf community rejects the label of disability*.”

### Career path of disabled academic researchers

After a diagnosis, several participants made changes to their research—seven specifically refocused onto disability-related topics. For some, this was entirely practical, as the physical environment or demands of their work were not accessible or accommodating. One participant described their transition towards new methods and said, *“I gave up my lab bench and that was a big*, *emotional track for me… That was hard and it happened very quickly*, *so in the same day I had brought that up*, *my lab bench was given to someone else*. *There wasn’t really time for that transition… [T]here’s no roadmap for how to do any of this*.*”* For others, this was a result of a new motivation towards disability research. One participant said, *“It’s been therapeutic for me to not be working so much [on] myself*, *but working on the problem in a broader scientific and cultural sense*.*”* The majority of participants gained new insight into their research, advocacy, and empathy for others as a result of their disability. One participant said, “*Disability has definitely influenced the focus of my research*, *my teaching and services activities*. *I feel more compassion for others with disabilities*, *people who need accommodations for various reasons*, *and I feel a call to advocacy*.*”* This allowed researchers to connect more with their participants and also motivated them to strive for cross-disability access and equity.

Thirty-four participants described disclosing their disabilities or chronic conditions to at least some colleagues or students. Participants having apparent disabilities expressed that they did not have a choice in whether to disclose their disabilities. The primary reason participants limited their disclosure was fear of negative consequences such as being perceived as less capable by peers and leadership, and many described the process of disclosure as stressful or uncomfortable. One participant said, *“I have been told to hide disabilities from PIs [Principal Investigators] so they don’t treat me differently*.*”* Many participants avoided disclosing their disabilities during the hiring process, primarily because they had concerns that they would be disqualified on the basis of assumptions made about their disabilities on the hiring side. Two individuals indicated concern that they would be seen as unobjective in their disability related research. One participant stated, *“Sometimes I think there’s a perception that researchers/academics with disabilities*, *especially those that disclose their disabilities*, *are only ‘advocates*,*’ do less rigorous research*, *or are only there to disrupt*. *And while I do think of myself as an advocate*, *I also think it makes my research stronger and more impactful*.*”* The reasons people chose to disclose were much more varied, however, including: to better connect with disabled students or colleagues, to advocate more strongly for disability equity, for their direct safety or accommodation request, or to be seen as disabled rather than incapable.

Many participants commented on the unique complexity of disclosing their disability in job and grant application processes. Two participants described being very open about their disability in job interviews so they could ask interviewers direct questions about disability inclusion at the potential workplace. However, several participants were unwilling to disclose that they had a disability in a job interview for fear of discrimination. However, of these participants, some were willing to disclose their disability on a grant application, especially for applications based on disability research; this was because they hoped disclosure would demonstrate lived experience and bolster their application.

### Ableism

Thirty-four of the participants raised the impact of ableism, stigma, and bias both at an institutional level and in professional relationships in academia. Participants commented on how leaving disability out of conversations around diversity, equity, and inclusion, constituted a form of exclusion at the organizational level. Networking was repeatedly noted as difficult, due to issues such as lack of masking to protect from disease such as COVID-19, hours that extend beyond 9 to 5, loud meeting areas, the exhaustion of travel, and more. At their own institutions, participants faced stigma from peers and leadership around their productivity, as one person stated, *“if someone takes leave for their own health*, *they are met with gossip*, *passive aggression and discrimination*.*”* Another participant said their superior suggested to them that their funding was acquired on the basis of their disability rather than merit. Many stated that bias prevented them from being able, or required them to work harder than others, to advance at a typical rate in their career, and in some cases led to people being pushed out of academia entirely. One person said, *“No one looks at my CV and recognizes that I was in incredible excruciating pain during this time and could not eat*. *Yet*, *despite that*, *I persevered*. *You don’t see that in my CV*.*”* One participant said they received lower teaching evaluations from students due to perceptions of their speech disability, and another reported, “*I was kicked out of my initial doctoral program when I was diagnosed with schizophrenia*, *and experienced significant disability-based discrimination in the doctoral program I graduated from*.*”*

These issues persisted at participants’ institutional disability service offices, where they found a lack of resources for researchers. One person stated that their experience was *“fully medicalized and overly dismissive of my actual … request*.*”* Several participants said that there is more attention to student access, with one person stating, *“over the course of my career in academia*, *it’s gotten progressively worse the further I get out from education*.*”* However, two participants found their institutions’ ADA attorneys helpful in navigating the accommodation process.

### Accommodation access

Twenty-two participants stated that they had used accommodations in the workplace. Much of this required work in advance to set up an accommodating environment, such as organizing an accessible schedule for writing and meetings, apps to track tasks, preparing accessible transportation, and creating a checklist for access to review before each class. Others happened in the moment, such as notetaking and lip reading when captioning was not available. Participants self-accommodated with a wide range of assistive technology in their work, including portable microphones in the classroom, dual monitor systems, automatic captions, screen reading software, braille display and notetakers, custom wheelchairs, white boards, and yellow tinted glasses.

Two people reported very positive, prompt provision of accommodations through official channels, though most had difficult experiences with delays or incorrect implementation. One hard of hearing individual said, after teaching in a classroom with poor acoustics, *“[T]hey were supposed to order these little microphones for me*. *This dragged on for months and months and months”*. Repeatedly, participants reported unsupportive leadership or administration. Another individual stated, *“No one is willing to change themselves for your disability*. *There’s accommodations*, *but they don’t transition into people giving you the accommodations*.*”* Participants said they experienced frequent neglect for their accommodation provision, whether the provisions were inconsistent, ignored, or forgotten, and that they were frustrated when left to manage their accommodations alone. One participant shared, *“I always thought that somehow there would be a system in place for helping that*, *but there isn’t*. *It’s all up to you and how well you navigate the system yourself*. *It’s very much you’re on your own*.*”*

One of the main reasons for delay were requirements for medical documentation of their disabilities. One participant describes the rigid process: *“The difficulty is a lot of medical situations don’t fit in the form because they require you to predict the future*. *There is no way to predict*, *essentially*, *a very unpredictable condition that knocks me out of practice for weeks at a time*. *There’s no way any accommodations process will do that*, *really*. *Everybody admits that*.*”* One participant described how getting documentation was difficult and used some discretionary account funds to pay for testing to find a diagnosis for their disability, *“There isn’t really support for figuring out*, *‘You’ve got this*, *and this is what this means*.*’ I think it will make me better at my job if I could be like*, *‘Yes*. *This is it*. *Therefore*, *these are the strategies that I do need to use*.*’”*

### Time management, sick leave, and work burden

Several participants, especially those with dynamic disabilities, or disabilities that impact people differently day to day, stated they experienced accessibility difficulties in adhering to deadlines and other time-based expectations. One participant said, *“The whole problem with academia is it presumes that you’re able to put in a 60-hour week*. *Anybody who can’t put in a 60-hour week*, *whether it’s because they have little children*, *or they’re pregnant*, *or they’ve got a medical issue*, *or they’ve got a relapsing*, *remitting diagnosis*, *whatever*, *we suddenly discover that we have to find that time somewhere*.*”* Additionally, some participants described facing discrimination when requesting sick leave. This environment left some participants unsure of any accommodations that could assist them. Many participants found working from home to be a helpful tool, one made more readily available during the COVID-19 pandemic. Working from home was beneficial for time flexibility and productivity, as it allowed people to avoid physical pain, to turn on automatic captioning on video communication programs, to avoid sensory overload, and to take breaks or rest as needed. One person shared, *“The pandemic gave me the accommodations the institution wouldn’t and showed how easy it can be to integrate people with similar disabilities*. *I can manage my health condition on my time scale at my house while being productive*, *joining meetings*, *and mentoring*.*”*

### Advocacy and community

Respondents found an overall lack of disability inclusion and low representation of people with disabilities in academia. A participant shared, *“I think that there are initiatives to improve [disability inclusion]*, *but I think that it is lacking*.*”* Participants found that they were underrepresented in diversity trainings and in academic positions, as one participant highlights: “*I regularly point out that a major weakness of our field is that enormous underrepresentation of direct experience among our ranks*. *It’s ridiculous that I’m regularly the only disabled person in a room*, *group*, *etc*. *focused on the study of people with disabilities*.*”* Another participant stated that these issues persist in publication and that “*publishers receive research about ableism and people with disabilities as very niche*,” stunting dissemination of the work.

Participants highlighted the importance of role models and mentors with disabilities, support systems, and community with other researchers with disabilities. The ability to share experiences and life trajectories not only constitutes a means of empowerment, but also helps in shaping career paths for the next generation of academics with disabilities. Several participants discussed mentoring students with disabilities, though this was typically unpaid labor. A participant stated, *“I use my experience with disability to provide a supportive and empathetic environment for students*, *primarily*.*”* Participants also felt supported by their students, as in the case of a participant whose students accommodated their visual disability by verbally describing tables and figures in group settings, *“For them I don’t often have to remind them or even ask them*. *Oftentimes they’ll ask me first*. *They’ll be like how can I do this best for you*? *What do you need*? *That is such a great interaction to have when somebody’s just aware and automatically wants to be inclusive*.*”* Another individual found community across institutions, and shared, *“The peer mentorship group with other scientists with hearing loss has been instrumental in pushing for accommodations at our annual research conference and providing social networking opportunities for us*. *We have even worked together to publish papers describing how scientific organizations can better support their scientists with disabilities*.*”* The absence of fellow disabled researchers in some instances led to difficulties in navigating disability-specific challenges in academia and a sense of isolation. One participant said, *“I’ve never had a mentor with a disability and generally find these challenges to be rather isolating to navigate alone”*.

In the face of extensive barriers, participants self-advocated for their access needs and understanding of their disability in academia. Three individuals discussed self-advocacy in advance or absence of negative experiences, such as for a blind professor who educated their students how to indicate they have a question or comment at the start of the semester. Self-advocacy also took place after access was denied, and participants highlighted that this was excessively time consuming of time intended for academics, and that this had to occur outside “official channels.” One person shared, *“I have to constantly advocate for myself*… *It’s been really hard to do that*. *It makes me really angry and frustrated*. *It takes up a lot of time*, *and it’s tiring*.*”* This participant, along with three others, referred to disability rights laws such as the ADA when self-advocating to colleagues and leadership, when seeking accountability, and when protecting against unlawful intrusive questions about a disability.

Many participants engaged in disability advocacy that extended beyond themselves into their department, institution, community, and beyond. One person stated, *“I don’t want this just fixed for me*. *I want this fixed for everybody*.*”* Aims differed across participants, as some wanted disability inclusion in existing systems and recognition of disability as diversity, while others wanted systemic change that was “transformative” or created a “paradigm shift.” Respondents did this formally in leadership positions focused on diversity and access, as a part of their research, within disability advocacy groups, in public talks, as well as outside typical role requirements.

## Discussion

Our qualitative analysis underscores the pervasive obstacles faced by researchers with disabilities in academia. Our results reinforce previous research showing that researchers with disabilities encounter systematic barriers characterized by unaccommodating communication and physical environments and structures historically rooted in bias and ableism, highlighting the importance of framing disability disparities through a social model framework [23]. We further highlight how considerations around disability identity and disclosure are shaped by peers’ perceptions and fear of stigma and discrimination.

A study shows that disabled academics face discrimination and harassment at twice the rate of their nondisabled peers, a problem that compounds for people with multiple marginalized identities, such as LGBTQ people, Indigenous people, and women [32]. Consequently, ableism impacts professional relationships between researchers with disabilities and students, peers, and leadership. Participants of this study reported experiences of social exclusion and marginalization when interacting with superior or peers, as also found in previous work [33]. Negative attitudes and discrimination against disabled researchers hinder professional success, translate into barriers to securing and maintaining job positions, and lead to an attrition of disabled academics. Institutions’ views of disability as a medical construct limits the growth of researchers with disabilities and places the responsibility of access solely on the individual with a disability [34, 35]. Additionally, the frequent exclusion of disability from diversity discussions and initiatives not only perpetuates ableist institutional practices, but precludes the possibility of change towards more accommodating and inclusive higher education environments [36]. There is a need to build stronger relationships between disabled researchers and their broader institutions through meaningful attitudinal and access changes as informed by people with disabilities’ lived experiences [32]. Intersectional approaches are critical to these changes, as disability is more prevalent among marginalized populations such as people of color, older adults, gender and sexual minorities, as well as people from lower educational attainment and income [37, 38]. University leaders should raise awareness of ableism among their leadership, faculty and staff, as well as create platforms for disabled academics across backgrounds to develop supports for this community.

Though Title 1 of the ADA requires employers to provide reasonable accommodation to employees with disabilities, inaccessibility and lack of disability inclusion are common to disabled researchers in the workforce and students [9]. In addition to shared challenges, disabled postsecondary educators often lack accessible resources and must command authority in classrooms not designed for people with disabilities [39]. Navigating accommodations is particularly challenging for researchers with disabilities in the workforce as institutions often lack centralized disability resource centers, require lengthy processes for documenting a disability, and are sometimes unable to provide accommodations needed. At present, disability resource centers are often limited to students, leaving researchers to figure out accommodations on their own while dealing with unsupportive leadership or administrators. Participants stressed the experience of stigma when requesting and providing evidence for accommodations, and frustration due to inflexible processes and reluctancy from institutions to provide accommodations, issues documented by previous studies [40, 41]. The Equal Employment Opportunity Commission (EEOC) regulates Title I of the ADA instead of the Department of Justice, with the former specializing on employment-related issues such as employment discrimination, and the latter having a broader mandate involving civil rights issues. While the existing regulatory framework allows employers to ask for reasonable medical documentation demonstrating an employee’s disability before providing a relevant reasonable accommodation, legal scholar Katherine MacFarlane criticizes this because it “*betrays the social model of disability on which the ADA rests and is inconsistent with legislative history and the EEOC’s own interactive process guidance*” [42]. Existing policies on accommodations should be enforced, and universities should develop efforts to raise standards beyond legal compliance to promote accessibility for all.

In light of the identified barriers, participants frequently discussed how they engaged in advocacy to drive change, built community to help others navigate accommodations, and understood the importance of role models to foster the participation of people with disabilities in higher education. Due to low representation of intersectional minorities in academia, previous research shows the difficulties faced by individuals when they, beyond navigating obstacles of a system unaccommodating to their identities, constitute the only source of support and mentoring for those sharing the same identity [43]. Our study aligns with previous research in highlighting how pivotal research and staff networks are as resources for peer-support and advocacy for scientists with disabilities [44]. Furthermore, to truly advance a culture of inclusiveness, it is key that academic and research institutions are proactive about learning from and elevating not only the experiences of disabled researchers, but of the larger community of people with disabilities to ensure allyship and create welcoming spaces for disabled individuals.

Our study is a reminder of the need for improved data collection on the experiences of researchers with disabilities, which are crucial for developing evidence-based approaches. This includes data that is longitudinal and disaggregated by racial and gender minority status, institution type, and career stage. The collection of these data could help identify institutions that have achieved measurable progress in improving the success and inclusion of disabled researchers. There is also need for harmonized databases across federal and state agencies collecting data on people with disabilities in educational institutions, and institutions should be incentivized to create centralized disability departments to expedite accommodations for researchers. A study using a novel university disability inclusion score highlighted that 60% of the top institutional recipients of NIH funding underperformed in inclusion and accessibility of their undergraduate programs [4]. Moreover, given the importance of building networks in academia, strategies to make scientific meetings and conferences more accessible should be scaled-up to ensure increased participation of disabled researchers and facilitate the establishment of meaningful connections that could provide opportunities for mentoring, scientific collaborations, and peer-to-peer support [45].

This study has important limitations. As for most qualitative studies, our sample was not representative of individuals by disability types, gender, race and ethnicity, or geographic location. Therefore, these results may not generalize across these groups and settings. Additionally, the small sample size did not allow for an examination of obstacles across demographic subgroups and STEM vs. non-STEM fields. This study primarily focused on individuals who were currently working as researchers in U.S. academic settings, though future work should consider the experiences of individuals who ultimately left research or academic institutions for other sectors or unemployment [5, 46]. Questions from the screening questionnaire, particularly the one on type of disability, were open-ended. While this gave participants the opportunity to self-identify as having a certain disability, the data does not allow us to make granular comparisons as these categories differed from the ones used in previous studies. Strengths of this study should also be noted, such as the prioritization of accessibility in every step of the data collection process, and giving respondents flexibility to either engage in live or written interviews, which supports inclusion of participants across a diversity of disability groups. Additionally, our interviewers had lived experiences as people with disabilities, and this commonality has been shown to promote trust with participants. We also followed an approach of *centering disability*, which places disability as an integral part of the interview process that fosters flexibility and innovation [47, 48].

## Conclusion

This study contributes to our understanding of the experiences of researchers with disabilities in academia in the U.S. A lack of research and limited acknowledgement of obstacles impacting this community has held back changes, both at the institutional and government level, precluding the improvement of inclusion of disabled researchers. As Dunn points out, disability continues to be an afterthought at academic institutions, and while there has been progress in making higher education institutions more accessible and inclusive of people with disabilities, institutions are slow to remove physical and structural barriers [49]. As a result, the overall experience of being disabled and working in academia is a tolling and complex undertaking that further deepens the already existing disparities for people with disabilities at higher education institutions, which perpetuates inequities stemming from reduced opportunities for participation in society.

Our results highlight that academic institutions have much work ahead to ensure researchers with disabilities are provided with equitable opportunities, resources, and supportive environments. Academic leadership must allocate resources to address and mitigate ableism, create more inclusive environments, and raise standards beyond the low bar of ADA compliance to promote equity, inclusion and belonging of disabled researchers.

## Supporting information

S1 Table Interview guide. (DOCX)

S2 Table Major themes and codes. (DOCX)

S1 Checklist Completed COREQ checklist. (PDF)
