# Supplementary material for: Experiences of researchers with disabilities at academic institutions in the United States
Source: PLoS One. 2024 Aug 15;19(8):e0299612. doi: 10.1371/journal.pone.0299612 (PMC11326606; doi:10.1371/journal.pone.0299612)
Supplement: S1 Table — (DOCX) [file pone.0299612.s001.docx]

**S1 Table. Interview Guide.**

| **Question** |
| --- |
| Is your disability/medical condition congenital or acquired? |
| When was the onset of your disability/medical condition? |
| If you identify as a person with disability (or disabled person), do you remember when you start to identify as such? (Please skip this question if you do not identify) |
| Is your disability/medical condition visible, non-visible, or somewhere in the middle (can be visible or non-visible at different occasions)? |
| What is your experience like as a person with disability pursuing a career in academia? |
| Does disability (or medical condition) play a role in your career trajectory? For example, does disability have an influence on your career success, promotion, publication, grant application, mentorship, teaching, research, service, social engagement/networking, etc.? |
| Does disability play a role in your interactions with other people at your current institution? (people here include faculty colleagues, staff members, students, administrators, leadership, etc.) If it does, what role does it play? |
| Does disability play a role in your interactions with people in your field? (People here include external reviewers, colleagues from other institutions, people in similar professional associations, research participants, etc.) If it does, what role does disability play? |
| Can you share with us about disclosure of your disability/medical condition at your workplace?  For example, 1) If your disability/medical condition is not visible, do you disclose your disability in different occasions: when you apply for jobs, apply for research grants, seeking accommodations in parking/transportation/teaching, when you are interacting with colleagues, students, etc.?  2) Why do you (or not) disclose your disability in these occasions? |
| Do you require disability accommodation(s) at work?  1) If you do, how was the experience of requesting and getting accommodation(s) (this can also include non-academic accommodations, such as parking, social gatherings, etc.)?  2) If you do not require accommodations, how do you cope with your disability/medical condition at work? |
| How do you feel about disability inclusion in your workplace and in your field? If possible, can you give some examples? |
| Can you share the impact of the current pandemic on you as a faculty/scientist with disability (or disabled faculty/scientist) working in academia? The impact can be positive and/or negative. |
| Is there anything else that you would like to share that we might not have covered in this survey? |
| Would it be okay if we follow up with you for any clarifications of your answers to the survey questions? |
