# Supplementary material for: Experiences of researchers with disabilities at academic institutions in the United States
Source: PLoS One. 2024 Aug 15;19(8):e0299612. doi: 10.1371/journal.pone.0299612 (PMC11326606; doi:10.1371/journal.pone.0299612)
Supplement: S2 Table — (DOCX) [file pone.0299612.s002.docx]

**S2 Table. Major themes and codes**

| **Theme** | **Code** | **Quote** |
| --- | --- | --- |
|  |  |  |
| **Identity, Visibility, and Disclosure** | objectivity | Very weirdly, medicine is really bad at acknowledging that researchers themselves are living with diagnoses, whether they're disabling or not. …It's a very tricky line to walk because we want to be objective as researchers. |
|  | identity | During my PhD program, I was teaching disability studies and that was when I really went downhill and had multiple surgeries and things like that. I think I was dealing with a lot of identity issues at that point. Who am I? How do I identify as someone with chronic illness? I do think that almost teaching in disability studies helped me come to terms with some of that. |
|  | multiple disabilities | The first questions were challenging as one disability is obvious (visual), the other is not (health). I have tried to address the differences I have experienced with these disabilities as I think it important to see that they are not always similar. |
|  | able persona | I do not generally disclose my disability in formal academic contexts [because] I don't want to be known for that - I'd rather be known for my work, and by making that invisible disability visible, I'm afraid I'd get a reputation of appropriating disability accommodations that aren't deserved. |
|  | visibility | On Zoom you might not know that I have any disability if I'm just having captioning and participating. Definitely when I'm in person in my workplace, I'm very visually disabled. I use a white cane. I wear hearing aids that are very visible usually. |
| **Career Path of Disabled Academic Researchers** | hiring | I encountered an explicitly ableist dean in my first interview as a faculty member. I'm thankful that the interviews were set up as a series of individual meetings with different people on the search committee. |
|  | academic positions | Deep ableism and my disability have definitely changed my career trajectory. I should have had tenure. I should already have tenure by all reasonable measures with what I'm capable of, but my body works at a slower pace, and the system punishes that severely. |
|  | confidence and agency | I do wonder how I managed clinically as a nurse sometimes 'cause I didn't know I was dyslexic. How did I do drug rounds? I wouldn't have become a nurse if I'd known I was dyslexic before. I don't think I'd have felt safe, and I wouldn't go back because of that. I've lost confidence. |
|  | career or lifestyle change | I had planned to go into academia but not when I did. That was definitely brought about because I physically couldn't continue clinical nursing. |
|  | funding | Funders don't care either. Don't even get me started on NIH. There is no investment in disabled researchers/PIs. NIH taking an explicit stand, leading and advocating, featuring researchers with disabilities, mandating disability inclusion in disability-related projects would help so much. But I don't see the dial moving even the tiniest bit. |
|  | grants | At least in my field a lot of [grants] have grant applications that are housed on online apps. Usually, most of the time, they're not screen reader accessible. You can't even make the font size bigger. Just like really static web pages that are a little outdated. … Then with the format of the application. Sometimes they allow for uploading or copy and pasting in the content of your grant. That doesn't allow for alt text or a data display or for a figure.... It causes real problems and hiccups. |
|  | research focus shift | My experience has been pretty difficult since I developed this condition. I did become a full professor several years into this. It has definitely changed the research that I do because the research that I basically got tenure for is no longer something I can easily do. It mostly had a professional impact because of the changes I had to make in my research trajectory. |
|  | disability insights from lived experiences | I use my experience with disability to provide a supportive and empathetic environment for students. |
|  | disclosure | It's frustrating when I disclose my disability as it is most often dismissed/diminished by colleagues who really don't understand how incredibly challenging it is sometimes to function. |
|  | networking | My biggest problem is non-work related, forced socialization with work colleagues (happy hours, lunches). I try to avoid crowds and have multiple dietary restrictions. If I avoid these events, I am branded "not a team player". If I go to these events and don't eat or bring my own food, I get bullied for not eating food that will make me sick. … Because these events are not technically part of the job, it's hard to request an accommodation and the accommodation is not really an accommodation, it's just "be nice to your co-workers." |
| **Ableism** | accessibility of communication | I usually anticipate that materials people give me will be inaccessible. Whether that's PowerPoint or a grant application, it's prob'ly gonna take me more time to access it than it would if they just emailed me an accessible version. I try to count for that in my workday. |
|  | accessibility of infrastructure and transportation. | People often forget that I am limited in what parts of campus I can access (our campus is wildly inaccessible). |
|  | accessibility in academic environment | I worked in industry with my disability and was very open about it and had no problem in industry, but in academia, it's very almost shameful. |
|  | disability awareness | Hearing loss makes things more challenging, especially as fellow academics do not always understand the impact of hearing loss |
|  | discrimination | It's an extremely competitive market, and I believe many academic employers (like many employers, generally) may discriminate against candidates with disabilities explicitly or implicitly because they perceive these candidates may require more support or produce less. |
|  | stigma | There's a lot of shame associated with mental health conditions, particularly, for professionals that are supposed to be keeping it all together. |
|  | disability inclusion | We have a DEI office and I asked them if they would consider putting the disability statistics on their webpage, but they told me that… the DEI office was only for underrepresented groups like minority, LGBTQ, and disability didn't count. Yeah, they let me know very openly that they don't consider disability something worth accommodating, or reporting, or fixing. |
|  | internalized ableism | There were a couple of instances that made me really accept that I was a person of disabilities...but I never wanted to own that. I think a lot of people with disabilities feel that way, especially with invisible disabilities. There's that internalized ableism that's so damaging, but particularly over the pandemic, finding a place to park, I had to get a disability placard. I think that in a way solidified how I felt as a person with disabilities because I needed it. |
|  | accountability | I don't see anyone being held accountable for not doing what's required and necessary related to disabilities. |
| **Advocacy and Community** | community | I've also discovered other scientists who are like me, and we have formed a peer-mentorship network which has been instrumental for feeling included and validated in academics. I also feel a sense of commitment for other trainees with disabilities and want to mentor and support them as they also go through the academic career trajectory. |
|  | mentorship | I've never had a mentor with a disability and generally find these challenges to be rather isolating to navigate alone. |
|  | support | The forms, and the paperwork, and the dates, and the numbers, and sometimes—yeah, organizing things that have to be done, certain repetitive things. I really struggle to get those right sometimes. I have lovely colleagues and friends who help me out. Otherwise, I don't know how—I don't know if I could do it. |
|  | legal engagement | During the interviews for my position one professor asked me how much will it cost for interpreting services. I told him right away that's an illegal question under the Americans with Disabilities Act. He backed way up. I put him in his place. |
|  | partnerships | I had to give up on... ever being a PI because my medical situation was so chaotic that the idea of being in a grant proposal writing mill, it's pretty hard even if you're not sick. If you have a very unpredictable medical condition that throws you out of commission for weeks at a time, it's impossible. I gave up on PI-ing, but I am still a co-PI. I basically have partnerships. I've been able to cobble together networks so that I'm still able to get done what I want. |
|  | self-advocacy | I have to constantly advocate for myself as people have to do. It's been really hard to do that. It makes me really angry and frustrated. It takes up a lot of time, and it's tiring |
|  | disability ingenuity | I think it takes a lot of resourcefulness to figure out what accommodations I need because there are not very many blind academics. |
|  | representation | One thing is that people with disabilities are definitely very much underrepresented in academia. I have always been the only blind PhD student, or the only blind postdoc, or faculty member at my institution, so I think that could potentially create a lot of social isolation. |
|  | disability advocacy | I have taken an outspoken, leadership role in disability access and inclusion by speaking at public events, by accepting to chair a university committee on disability access and inclusion, etc. |
| **Accommodation Access** | assistive technologies | My university has purchased a few pieces of assistive technology for me that has been really helpful for me to carry out my work. |
|  | disability documentation | The accommodation process was very splintered between HR and Occupational Health. They kept asking for multiple versions of the same accommodation paperwork, like letters from my same physicians over and over again. It was really burdensome and took almost eight months I think once I started working to get everything that I needed. |
|  | access to care | When I was first diagnosed, my faculty appointment gained me more ready access to treatment and follow-up than I would of had in many other jobs . |
|  | disability services | The accommodation office seems generally confrontational even though clearly my work did not suffer from having to do it remotely. |
|  | accommodation neglect for researchers | I had the forethought to include my accommodations in my contract with the university, and I still have challenges getting my accommodations fulfilled. |
|  | disability-related expenses | Many academic institutions, the first thing that they think of when a faculty with a disability asks for accommodations is, quote, "how much is it gonna cost us." |
|  | work from home | That everyone switched to full remote (at least for a while) made me feel less marginalized... however, as the rest of campus/ society "opens up" and drops mitigations, the immunocompromised are really left behind. |
| **Time Management, Sick Leave, and Work Burden** | time and flexibility in timelines | I bank on not being at work before nine a.m., if only because I still am coping with a lot of the fatigue in the mornings. It takes a lot of my energy to get up and get my son ready for school, along with my husband. Most of my critical care colleagues are in by 6:30 or 7, and there's just no way I would ever be able to do that. There are some accommodations that aren't explicit, but the flexibility of my job allows them. |
|  | deadlines | My major experience as it relates to my academia (and life in general) is there are periods of time when it's really hard to get work done, due to pain, fatigue, and brain fog. Sometimes there is just a single day where I can't work but other times the period lasts weeks or even months and it becomes disabling. It's hard to figure out expectations and communicate revised deadlines with people because I myself don't know when a flare up is going to end. |
|  | work burden | It feels like I have to work harder and longer to stay on pace with colleagues |
|  | sick leave | I have an implanted device for my stomach, and the battery died four weeks after I started my new job. I needed emergency surgery and was gonna be out for a bit. I had to disclose because I didn't have enough sick leave built up yet. I needed to take time off and tell them what was going on. |
